# RETRACTION: Proinflammatory Cytokines IL‐6 and TNF‐α Increased Telomerase Activity through NF‐κB/STAT1/STAT3 Activation, and Withaferin A Inhibited the Signaling in Colorectal Cancer Cells

**DOI:** 10.1155/mi/9868413

**Published:** 2026-01-09

**Authors:** Mediators of Inflammation

RETRACTION: S. S. Chung, Y. Wu, Q. Okobi, D. Adekoya, M. Atefi, O. Clarke, P. Dutta, and J. V. Vadgama, “Proinflammatory Cytokines IL‐6 and TNF‐α Increased Telomerase Activity through NF‐κB/STAT1/STAT3 Activation, and Withaferin A Inhibited the Signaling in Colorectal Cancer Cells,” *Mediators of Inflammation* 2017: 5958429, https://doi.org/10.1155/2017/5958429.

The above article, published online on 06 June 2017 in Wiley Online Library (wileyonlinelibrary.com), has been retracted by agreement between the journal Editor‐in‐Chief, Anshu Agrawal, and John Wiley & Sons Ltd. The retraction has been agreed due to concerns identified with the Figures 1, 2 and 6 on PubPeer [1]. On further investigation, additional concerns were identified with multiple Western blots. A summary of the concerns are as follows:

• In the HT‐29 right panel of Figure 1a, the STAT3 blots appear identical to the IP: STAT1 blots shown in Figure 6c.
• In Figure 2b, the fourth STAT3 band appears identical to the STAT3 band shown in Figure 2a.
• In Figure 2c, the NF‐kB band appears similar to the STAT3 band after a horizontal flip.
• In Figure 6b, the first two IB: STAT1 bands appear identical to the first two bands shown in the IP: STAT1 bands shown in Figure 6c after 180 degree rotation.
• Multiple blots within the paper show evidence of undeclared splicing.

Following evaluation of the concerns and authors’ response, the editorial board has determined that the results and conclusions could not be considered reliable, and the article is retracted.

Debbie Adekoya agrees to the retraction. The remaining authors did not respond to the retraction.
